# P07-02 Prescription of adapted physical activity: expectations of patients consulting general practitioners in the department of Yvelines

**DOI:** 10.1093/eurpub/ckac095.102

**Published:** 2022-08-29

**Authors:** Christophe Kanso, Jean-Christophe Blanchard, Alexis Astruc

**Affiliations:** 1 Université de Versailles Saint-Quentin en Yvelines, Montigny-Le-Bretonneux, France; 2 Ellasanté Health Center, Paris, France; 3 Department of General Practice - University Paris 13, Bobigny, France

**Keywords:** physical activity prescription, patient, primary care, general practice

## Abstract

**Background:**

Since 2016, French law has allowed general practitioners to prescribe adapted physical activity (APA) to patients with long-term health conditions. Studies have demonstrated that physicians are interested in this practice but very few have surveyed patient expectations.

**Objective:**

The objective of this work was to study patients' expectations regarding the prescription of APA.

**Methods:**

This is a quantitative descriptive study treating data obtained from a survey carried out in general practice offices in the department of Yvelines between June and September 2019. The inclusion criteria for patients were: above 18 years old and present in the waiting room.

**Results:**

252 patients were surveyed in 9 doctors' offices. We received 90% of responses. Prescription of APA is a good idea for 95.2% of patients, 80.2% were motivated to join an APA program and 67.4% thought that prescription of APA would increase their motivation. The main constraints to physical activity practice were lack of time (59.9%) and lack of motivation (31.7%). The main practices that would help patients to better adhere to an APA program were group sessions supervised by a professional (64.7%) pursued by a follow up with their doctor (41.7%). 53.2% of patients considered that it would be important to monitor their physical activity through a connected device. Factors independently associated with the motivation to participate in the APA program were a female gender (*p* > 0.01), a bachelor's degree (*p* = 0.01), a general opinion that prescription of physical activity is a good idea (*p* > 0.01).

**Conclusion:**

Our results revealed an enthusiasm with patients for APA prescription, which is interesting for the promotion of PA in general medicine.

